# Supplementary figures and images for: Time-frequency analysis reveals an association between the specific nuclear magnetic resonance (NMR) signal properties of serum samples and arteriosclerotic lesion progression in a diabetes mouse model
Source: PLoS One. 2024 Mar 8;19(3):e0299641. doi: 10.1371/journal.pone.0299641 (PMC10923453; doi:10.1371/journal.pone.0299641)

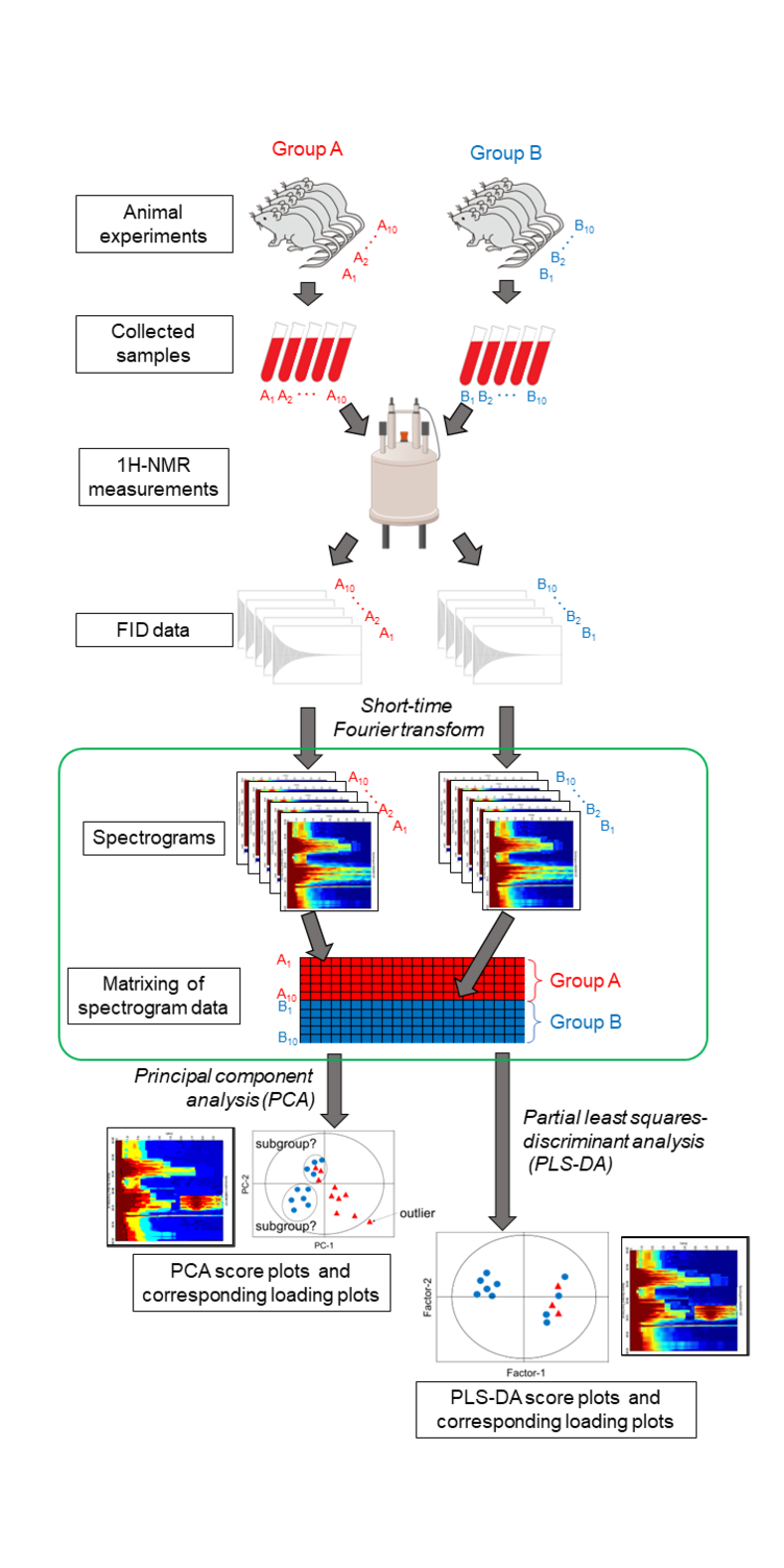

Supplement: S1 Fig — In this figure, the results of NMR analysis of blood samples collected after animal experiments in groups A (n = 10) and B (n = 10) are shown. In total, 20 1H-NMR FID signal data were acquired, which were short-time Fourier transformed [51], and 20 spectrograms were calculated. For multivariate analysis, a process was performed to obtain a data matrix from the 10 spectrograms. From this data matrix, an exploratory analysis was performed using principal component analysis [26] for the identification of outliers and potential subgroups of animals in groups A and B. From this figure, it was possible to identify subgroups that were not initially anticipated, so PLS-DA [52, 53] was performed only with data that corresponded to subgroups in the same data matrix to analyze whether subgroups could be identified. In both principal component analysis and PLS-DA, the variables that were important for the identification of groups in each analysis were plotted as corresponding score plots. This score plot has the same time and frequency axes as the spectrogram and thus can be compared with the spectrogram. The illustration of the mouse and the NMR equipment in this figure was obtained from Research Net, a website where research illustration material is available free of charge (https://www.wdb.com/kenq/illust © WDB Co., Ltd., Tokyo, Japan). References (cited only in the Supporting Information) 51. Wacker M, Witte H. Time-frequency techniques in biomedical signal analysis. a tutorial review of similarities and differences. Methods Inf Med. 2013;52:279–296. 52. Barker M, Rayens W. Partial least squares for discrimination. J Chemo. 2003;17:166–173. 53. Sylvie Chevallier S, Bertrand D, Kohler A, Courcoux P. Application of PLS-DA in multivariate image analysis. J Chemo. 2006;20: 221–229. (TIF) [file pone.0299641.s001.tif]

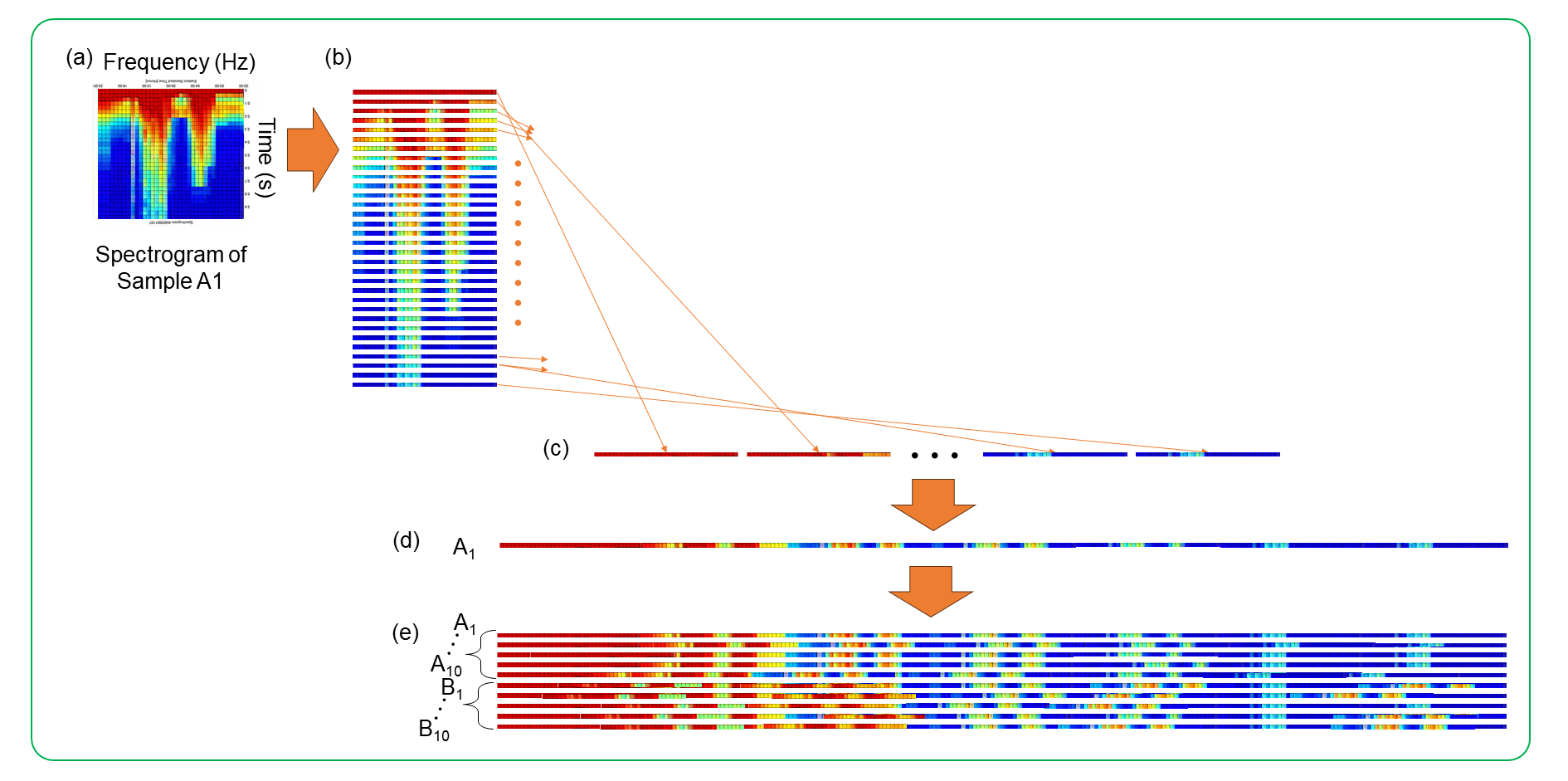

Supplement: S2 Fig — The process surrounded by the green box in S1 Fig is described in detail. For multivariate analysis of spectrogram data, a 256 × 1024 spectrogram (a) with frequency on the horizontal axis and time on the vertical axis was divided into 1024 single rows (b). All rows were re-arranged into a single row, and 1 × 262,144 single rows were reshaped (c). Since a single row represents spectrogram data for one individual (d), the single rows of spectrogram data for all individuals needed for multivariate analysis are combined to create a data matrix (e). (TIF) [file pone.0299641.s002.tif]
