# Supplementary material for: Time-frequency analysis reveals an association between the specific nuclear magnetic resonance (NMR) signal properties of serum samples and arteriosclerotic lesion progression in a diabetes mouse model
Source: PLoS One. 2024 Mar 8;19(3):e0299641. doi: 10.1371/journal.pone.0299641 (PMC10923453; doi:10.1371/journal.pone.0299641)
Supplement: Attachment — Submitted filename: ResponseToReviewers.docx [file pone.0299641.s004.docx]

**Reviewer #1: General Comments**
In this study, the authors analyzed serum sample from diabetic model mice (BKS. Cg db/db) by NMR and compared the results with the development of atherosclerosis observed in carotid artery samples. They concluded that NMR modal properties of serum are associated with arteriosclerotic lesion. They also used control mice (Jc1:ICR) without diabetes and checked their carotid arteries and lipid profiles.

**Specific comments**
1. Serum glucose in diabetic model mice is much higher than in control mice, and in addition to hyperglycemia, there appear to be changes in inflammatory markers and hormones. Can you say that the NMR findings are reflected in changes in carotid intima thickness and atheroma rather than these changes in blood? The reviewer thinks more logical evidence is needed to make NMR information from blood reflect arteriosclerosis.
>Response: Thank you for providing these insights. As pointed out by Reviewer 1, we consider that there are also inflammatory markers and hormonal fluctuations associated with the onset and progression of hyperglycemia and carotid atherosclerosis. Therefore, we consider the NMR mode characteristics of the diabetic mouse serum model presented in this study to include information on inflammatory markers and hormonal fluctuations associated with hyperglycemia and carotid atherosclerosis.

For blood glucose in serum, see Table 2 [Author’s Note. Revised Table 2 when Tables 1-3 are combined] in which the diabetic model mice show significantly higher results than the control mice. However, if we look at the diabetic model mice, the results do not show a clear increase in blood glucose levels with an increasing number of weeks. Therefore, given that our results are only for the diabetic model group, we consider the NMR findings are largely unaffected by hyperglycemia.

The results of the BS in revised Table 2 are represented graphically and attached as a reference figure for the reviewers.

The aim of our study was to investigate whether the NMR modal properties of serum, which are affected by these inflammatory markers and hormonal changes, are associated with carotid atherosclerosis. Serum is a complex mixture containing various metabolites, ionic small molecules, and albumin, and we believe that NMR modal analysis can be used to evaluate the interactions among these substances as well. The elucidation of disease-specific inflammatory markers and hormonal fluctuations is an important topic in bioscience. In the same way, if disease-specific NMR mode characteristics of serum could be clarified, it would be of great diagnostic significance. We recognize that this study has made it possible to identify NMR mode properties associated with carotid atherosclerosis from the serum of diabetic mouse models.
To describe this logically and fully, we have added the following text to the Discussion section.

pp.17, lines 381-385. The following sentence has been added: “It should be noted that since atherosclerotic lesions are associated with certain biochemical changes in serum (inflammatory markers and hormones), the modal properties of this serum NMR should be considered to include inflammatory markers and hormonal changes associated with the progression of atherosclerotic lesions.”

2. Why did we examine changes in arteriosclerosis only in the carotid artery? The aorta is easier to study and may be more relevant to serum changes due to its larger area.
>Response: Thank you for your question. This study was conceived with the idea that if there was a way to predict carotid artery stenosis, which is a contributing factor to stroke, a blood test could be used to help manage the risk. The mouse carotid artery has a higher percentage of smooth muscle cells than the mouse abdominal aorta, confirming the morphological characteristics of muscular arteries. In this study, the mouse carotid artery was selected as a model experiment for the human carotid artery. The following text has been added to the legend of Figure 1 to help the reader understand the histology of the carotid artery.

pp.12, lines 252-253. The following sentence has been added: “…showing a normal histology, with the tunica media composed of numerous smooth muscle cells.”

3. Why is there no NMR mode analysis of serum from control mice (JC1:ICR)? Shouldn't there be a comparison?
>Response: Thank you for your suggestion. We have already performed PCA analysis on serum samples from diabetic model mice and control group mice. We interpreted the results of this analysis as only representing the clustered NMR mode characteristics of the sera from the BKS and Jcl mouse groups. Given the purpose of this study, we have tried to make it easier for the reader to understand the results of the PCA by providing a brief "summary of the results of the PCA analysis including the control group". Thank you for your valuable comments and suggestions.

pp.13, lines 267-268. The following sentence has been added: “The PCA on spectrogram data of all mouse serum samples showed that the BKS and control groups clustered in the PC1/PC2 score plots (data not shown).”

4. Tables 1, 2, and 3 contain the same information for each mouse, so it's a good idea to combine the three tables into one table.
>Response: Thanks for this excellent suggestion to integrate our tables. We followed the *PLOS ONE* guidelines and integrated Tables 1, 2, and 3 (pp.10, line 214 - pp.11, line 225).

pp.9, lines 204-206. The following text has been revised: “….lists the lesions observed at each week for each mouse of the Jcl:ICR and BKS.Cg db/db strains, except for mouse ID.B22-2, for which carotid artery samples could not be obtained at necropsy.”

pp.11, line 236. The table number has been revised: “Table 2 ….”

5. RE: Figure 1. Mice of a given age have different IMT and atheroma severity and lipid profiles, so please indicate from which mouse the carotid artery was harvested.
>Response: Thank you for your suggestion. We have added the ID of each individual mouse to the legend of Figure 1.

pp.12, line 252. The following subject ID has been added: (B10-1).
pp.12, line 254. The following subject ID has been added: (B14-4).
pp.12, line 255. The following subject ID has been added: (B18-5).
pp.12, line 257. The following subject ID has been added: (J18-1).

6. RE: Figure 3. Are red circles PC1/PC5 of BKS 18W, not BKS26W?

>Response: You are absolutely correct. There was a mistake in the key placement in Figure 3. The red circle is BKS 18W, not BKS 26W. Please accept our sincerest apologies.

7. RE: Figure 3. “In this figure, the PC1/PC5 score plot (Figure 3A) showed that groups B10 and B14 formed one loose cluster with similar PC1 scores.”
Data from the B14 group showed similar PC1 scores but variable PC5 scores. Your description is wrong.
>Response: You are absolutely correct. The incorrect key was included in our original Figure 3. Please accept our sincere apologies for this oversight.

8. RE: Figure 3. “PC1 scores were organized as follows: B26 group > B22 group > B10 and B14 groups.” Please indicate the average PC1 value for each group.
>Response: There was an error in the placement of the key in Figure 3, and there was a discrepancy between the figure and the description in the text. Please accept our apologies for this oversight.

9. Please distinguish BKS10W from BKS14 and BKS22W from BKS26W.
>Response: There was an error in the placement of the keys in Figure 3. We would like you to see the revised Figure 3 in which the key has been corrected.

10. RE: Multivariate analysis. “Variables with an absolute value ≥0.4 as the loading value were considered relevant.”
Clearly indicate where variables with an absolute value ≥0.4 as the loading value were considered relevant.
>Response: Thank you for your review of our manuscript. In a multivariate analysis, it is important to limit the range of absolute values of the relevant loading values. If the range is too wide, noise components may become over-extracted. On the other hand, if the range is too narrow, it may exclude regions that are useful for the analysis. In an exploratory study, this is not a statistically a priori determined value, but rather an exploratory determination of the range. Specifically, a discriminable range must be established for each individual study setting with reference to the NMR spectrograms and loading plots obtained. In this study, that was 0.4. In the revised manuscript, we have added text to help the reader understand the purpose of the value of 0.4.

pp.14, lines 287-289. The following text has been added to the Figure 3 legend: The threshold values, which were set in an exploratory manner based on the characteristics of the analyzed data to increase discriminability, were set above 0.4 and below −0.4.

11. RE: Figure 4. The value at the bottom of the horizontal axis not 150, but -150.

>Response: As you indicated, the "150" is incorrect. We had added a "-", but it was not displayed correctly. Please accept our sincere apologies for this oversight.

**Reviewer #2:** The authors clearly showed that NMR analysis using serum samples correlated severity of atherosclerosis in diabetic mice model. This study would be clinically useful for early detection for atherosclerosis progression using blood sample. And also the manuscript is well described about study protocol, design, and presentation of the results. However, some issues should be fixed for publication.

**General comment**
1. All animals were not used for each NMR analysis. The author should describe how selected each animal for each analysis or randomly chosen.

>Response: Thank you for your remarks. First, we performed a glucose tolerance test and blood glucose monitoring on a group of mice to determine their glycemic dynamics.

As a result, we estimated the age at which hyperglycemia is maintained to be between 10 and 26 weeks, so for another group of mice, we collected 4 BKS.Cg db/db mice at 10, 14, 22 and 26 weeks of age and the remaining 12 mice at 18 weeks of age. Biochemical tests were also performed on this group of mice (revised Table 2).

In the revised manuscript, the text has been modified as follows to clarify that the group of mice subjected to analysis of blood glucose dynamics is separate from the group of mice subjected to sample collection and biochemical testing.

pp.5, lines 100-101. In the following text, the underlined words have been inserted: “Glucose tolerance testing and blood glucose monitoring were performed on a set of 16 mice (4 mice each of the Jcl:ICR, C57BL/6JJcl, BKS.Cg db/db, and BKS.Cg -/db strains) during rearing to determine their blood glucose dynamics.”

pp.5, lines 116-117. In the following text, the underlined words have been inserted: “Samples were taken every 4 weeks for 26 weeks, starting at 10 weeks of age for BKS.Cg db/db mice, apart from the aforementioned set of mice.”

2. Each table and figure legend should be described altogether after the manuscript for easy to understanding the text.
>Response: Thank you for pointing this out. We have followed the *PLOS ONE* guidelines.

3. Author should explain each NMR probe in detail, such as PCA, PC-1, PC-5, PLS-DA, and so on.
>Response: Thank you for your review of our manuscript. The identification of NMR probes useful for discrimination in multivariate analysis results is of considerable importance. The NMR probe in NMR modal analysis can be regarded as an inherent signal property of the sample-derived NMR-FID signal. This signal property can be determined based on interactions with intra- and intermolecular protons, but these interactions are complex, making it difficult to accurately describe the NMR probe at this time. However, as shown in the loading plots in Figures 3-7, we have successfully visualized the serum-derived FID signal properties that are useful for identifying atherosclerosis.
Based on the above, we have revised our manuscript as follows.

pp.17, lines 376-379. In the following text, the underlined words have been inserted: "The correlation loading plots indicate which parts of the NMR modal properties are valid for discrimination. These plots can be interpreted as presenting a visualization of the parts of the overall properties of the serum sample that are relevant to the characteristics of the mouse model of atherosclerosis.”

**Specific comment**
1. About analysis of 18 weeks BKS mice, author should describe the relation between pathological findings (severity of atherosclerosis) and NMR results.
>Response: Thank you for mentioning this. As shown in Table 1, rapid progression of atherosclerosis in BKS mice is observed at 18 weeks. In the PCA score plot in Figure 3, the BKS mice were bisected into two groups: a group of mice with no or progressive atherosclerosis and younger than 18 weeks, and a group with progressive atherosclerosis and older than 18 weeks. However, the plot for the 18-week-old BKS mice was scattered.

The results of this score plot may reflect changes in serum NMR properties based on the degree of atherosclerosis. On the other hand, we interpret the results as suggesting that at 18 weeks of age, a time of rapid progression of carotid atherosclerosis, NMR modal properties may also be significantly affected by rapid changes in inflammatory markers and hormones associated with the progression of atherosclerosis. That is, serum NMR may accurately reflect not only pathological findings (severity of atherosclerosis) but also dynamic changes in pathological findings (severity of atherosclerosis). However, as our study is exploratory, it is premature to suggest that our results reflect the true nature of serum NMR in diabetic mice or to establish a diagnostic model based on the present results alone. Therefore, in the Discussion section, we have mentioned the discordance between the multivariate analysis results and pathological findings, and added the following statement.

pp.18, lines 393-397. In the following text, the underlined words have been inserted: “Second, there was some discordance between the multivariate analysis results and pathological findings of BKS.Cg db/db mice based on differences in the biological reactions in individual mice. The biological reactions may partly reflect the serum of mice with atherosclerosis. On the other hand, in 18-week-old mice with rapidly progressive atherosclerosis, the reaction may be associated with pathological progression. Unfortunately, our exploratory studies are limited in their ability to elucidate the true nature of this biological response and will be the subject of future research.”
