# Supplementary material for: Time-frequency analysis reveals an association between the specific nuclear magnetic resonance (NMR) signal properties of serum samples and arteriosclerotic lesion progression in a diabetes mouse model
Source: PLoS One. 2024 Mar 8;19(3):e0299641. doi: 10.1371/journal.pone.0299641 (PMC10923453; doi:10.1371/journal.pone.0299641)
Supplement: Attachment — Submitted filename: comments-plos one.docx [file pone.0299641.s005.docx]

The authors used NMR method to analyze serum samples of diabetic model mice and compared the results with the development of atherosclerosis observed in carotid artery samples. They concluded that revealed that NMR modal properties of serum are associated with arteriosclerotic lesions. Thus, it may be worth exploring the possibility that the risk of cardiovascular events in diabetic patients could be assessed using serum samples.

1. Authors should give more examples for the relation of disease or other pathological condition with NMR modes. What are the pathological mechanisms for these relation? Please mention “Nuclear magnetic resonance (NMR) is a phenomenon that occurs when the nuclei of certain atoms are moved from a static magnetic field to an oscillating magnetic field. Radiofrequency pulse at the resonance frequency causes impulse responses so called free induction decays (FIDs) during NMR measurements. By analyzing FIDs using Fourier transform (FT), we can obtain characteristic spectrums of substances (frequency analysis). Some investigators have studied the utility of this modality in discriminating serum taken from patients with cancers such as renal cell carcinoma, hepatocellular carcinoma and lung cancer” from the reference 18. These knowledge is important for readers to understand the topic.

2. “NMR modal analysis of serum is reported useful for the diagnosis of pancreatic cancer”, please explain how it is useful? Am agree the other reviwers that a direct relation seems not logical. On the other hand there may be indirect relation related to inflammatory markers and hormonal fluctuations associated with the onset and progression of hyperglycemia and carotid atherosclerosis

3. Authors said “We recognize that this study has made it possible to identify NMR mode properties associated with carotid atherosclerosis from the serum of diabetic Mouse models.”. Their answer is not based a scientific perspective. Please give more evidence and explanation that are supported with other publications or their hypothesis. Their hypothesis is very weak to understant this relationship.

Minor points

1. Free induction decay (FID should be indicate in the first use.
